# Anlotinib for Metastatic Progressed Pheochromocytoma and Paraganglioma: A Retrospective Study of Real-World Data

**DOI:** 10.1210/jendso/bvae061

**Published:** 2024-04-02

**Authors:** Rui Tian, Xiaochen Yao, Jieping Song, Jun Wang, Jingjing Fu, Liang Shi, Fei Yu, Pengjun Zhang, Chuan Zhang, Yudan Ni, Feng Wang

**Affiliations:** Department of Nuclear Medicine, Nanjing First Hospital, Nanjing Medical University, Nanjing 210006, China; Department of Nuclear Medicine, Nanjing First Hospital, Nanjing Medical University, Nanjing 210006, China; Department of Nuclear Medicine, Nanjing First Hospital, Nanjing Medical University, Nanjing 210006, China; Department of Nuclear Medicine, Nanjing First Hospital, Nanjing Medical University, Nanjing 210006, China; Department of Nuclear Medicine, Nanjing First Hospital, Nanjing Medical University, Nanjing 210006, China; Department of Nuclear Medicine, Nanjing First Hospital, Nanjing Medical University, Nanjing 210006, China; Department of Nuclear Medicine, Nanjing First Hospital, Nanjing Medical University, Nanjing 210006, China; Department of Nuclear Medicine, Nanjing First Hospital, Nanjing Medical University, Nanjing 210006, China; Department of Nuclear Medicine, Nanjing First Hospital, Nanjing Medical University, Nanjing 210006, China; Department of Nuclear Medicine, Nanjing First Hospital, Nanjing Medical University, Nanjing 210006, China; Department of Nuclear Medicine, Nanjing First Hospital, Nanjing Medical University, Nanjing 210006, China

**Keywords:** anlotinib, pheochromocytoma, paraganglioma, multi-kinase inhibitor, peptide receptor radionuclide therapy

## Abstract

**Introduction:**

Pheochromocytomas (PCC) and paragangliomas (PGL) (collectively PPGL) are a type of rare hypervascular neuroendocrine tumors that are very challenging to treat. This study aimed to determine the efficacy and safety of the multi-tyrosine kinase inhibitor anlotinib for the treatment of locally advanced or metastatic (LA/M) PPGL.

**Methods:**

A total of 37 eligible patients with unresectable or progressive LA/M PPGL were enrolled. Of them, 27 patients received anlotinib alone (n = 19) or in combination (n = 8) with radionuclide therapies, including peptide receptor radionuclide therapy (PRRT) and iodine 131 meta-iodobenzylguanidine (^131^I-MIBG). The primary endpoints included objective response rate (ORR), defined as partial response (PR) or complete response (CR), and disease-control rate, defined as PR, CR, or stable disease (SD). The secondary endpoints were progression-free survival (PFS), duration of response, and drug safety.

**Results:**

In the efficacy evaluation for all 27 patients, the ORR was 44.44% (95% CI: 24.4%-64.5%) and disease-control rate was 96.29% (95% CI: 88.7%-100%). Twelve cases (44.44%) achieved PR, 14 (51.85%) SD. The median PFS was 25.2 months (95% CI: 17.2 months to not reached). PFS was shorter in the anlotinib monotherapy group than in the group receiving anlotinib in combination with radionuclide therapy (*P* = .2). There were no serious treatment-related AEs.

**Conclusion:**

Anlotinib monotherapy or in combination with radionuclide therapies shows promising efficacy and safety for the treatment of LA/M PCC and PGL. Multi-tyrosine kinase inhibitors might represent a novel therapeutic strategy for patients with PPGL; however, large-scale prospective randomized, blinded, controlled clinical research studies are required.

Pheochromocytomas (PCC) and paragangliomas (PGL) (collectively PPGL) a type of rare hypervascular neuroendocrine tumors, are derived from either chromaffin cells in the adrenal medulla or the sympathetic or parasympathetic extra-adrenal paraganglia [1]. The prevalence of PPGL is approximately 2 to 8 people per million per year [2]. Epidemiological data are largely lacking in Chinese populations; however, the estimated incidence of epinephrine-producing PGL was higher in Chinese than in European populations, mostly due to the presence of *HRAS* and *FGFR1* mutations [3]. Sympathetic tumors frequently secrete excessive catecholamines, resulting in clinical syndromes, such as hypertension and palpitations, and even life-threatening cardiac complications, such as hypertension crisis, malignant arrhythmias, or cardiogenic shock. In contrast, tumors with less catecholamine-associated symptoms and/or signs have an insidious onset, which may delay their early diagnosis and treatment [4]. In addition to abovementioned cardiovascular complications, PPGLs confer different risks of malignancy, with 15% to 20% of the cases being metastatic [5, 6]. The prognosis of these tumors thus remains poor. Surgical resection should be the first-choice treatment for metastatic PPGLs [7]. Apart from high-specific activity [^131^I]-meta-iodobenzylguanidine (MIBG) in the United States [8], there are no generally approved systemic treatment options for metastatic PPGL, and few treatment options for patients with advanced PPGL. Targeted radionuclide therapies (eg, ^131^I-MIBG or ^177^Lu-DOTA-SSA therapy [peptide receptor radionuclide therapy; PRRT]) and chemotherapy (eg, cyclophosphamide, vincristine, and dacarbazine, or temozolomide monotherapy) have been reported for the treatment of PPGL in small populations or case reports [8-13]. In previous studies, however, the 5-year overall survival (OS) of patients with metastatic PPGL was only 30% to 60% [5, 14]. Mutations in the succinate dehydrogenase (SDHx) gene predispose to an aggressive phenotype in 70% of patients, resulting in distant metastasis, tumor multiplicity, and disease recurrence [15-18]. Given the unsatisfactory efficacies and limitations of current treatment options, some novel treatments for PPGL are urgently needed.

Higher vascular endothelial growth factor (VEGF) receptor expression was observed in metastatic PPGL, suggesting that VEGF-mediated angiogenesis may be related to PPGL tumor progression [19]. Tyrosine kinase inhibitors (TKIs) based on molecular pathways leading to tumorigenesis in PPGL have accordingly demonstrated antineoplastic effects; however, few studies have reported on the effects of TKIs, including sunitinib, pazopanib, and cabozantanib, in metastatic PPGL [11, 20-23], with the largest sample size in a study of 25 patients treated with sunitinib. Anlotinib is a novel multi-TKI targeting the VEGF receptor, platelet-derived growth factor receptor, fibroblast growth factor receptor, and c-Kit, and it has demonstrated efficacy against various malignant tumors, including advanced non-small cell lung cancer, soft-tissue sarcoma, and thyroid cancer [24-26]. PPGL exhibits similar changes in tumor pathology and biological behavior to these other tumors; however, there were no clinical trials evaluating the efficacy of anlotinib therapy for PPGL. We speculated that anlotinib could exert a potent antitumor effect on the locally advanced or metastatic (LA/M) PPGL.

In this preliminary study, we retrospectively analyzed patients treated with anlotinib monotherapy or in combination with radionuclide therapies for LA/M PPGL in our center, to investigate its efficacy and safety in a real-world setting. To the best of our knowledge, this is the first study to examine the efficacy and safety of anlotinib for the treatment of PPGL.

## Methods

### Patient Selection

This study adhered to the Declaration of Helsinki and was approved by the Ethics Committee of Nanjing First Hospital (approval number: KY20231109-01). The need for informed consent was waived by the ethics committee due to the retrospective nature of the study. The study enrolled patients with a histologically or cytologically confirmed diagnosis of locally advanced (unresectable) or metastatic PPGL, who were treated at Department of Nuclear Medicine, Nanjing First Hospital, China, from March 2020 to October 2021. The inclusion criteria of patients included: (i) aged > 18 years; (ii) diagnosed with evident radiological imaging by the response evaluation criteria in solid tumors (RECIST) version 1.1 criteria and/or biochemical abnormality; and (iii) new presence of the tumor-related symptoms but the absence of radiological progression evidence, which needed to be evaluated by 3 certified nuclear medicine physicians. Patients were excluded if they had severe dysfunction of their major organs, or antitumor therapy within 21 days before the study. The detailed inclusion and exclusion criteria are listed in Supplementary Material 1 [27].

**Figure 1.**
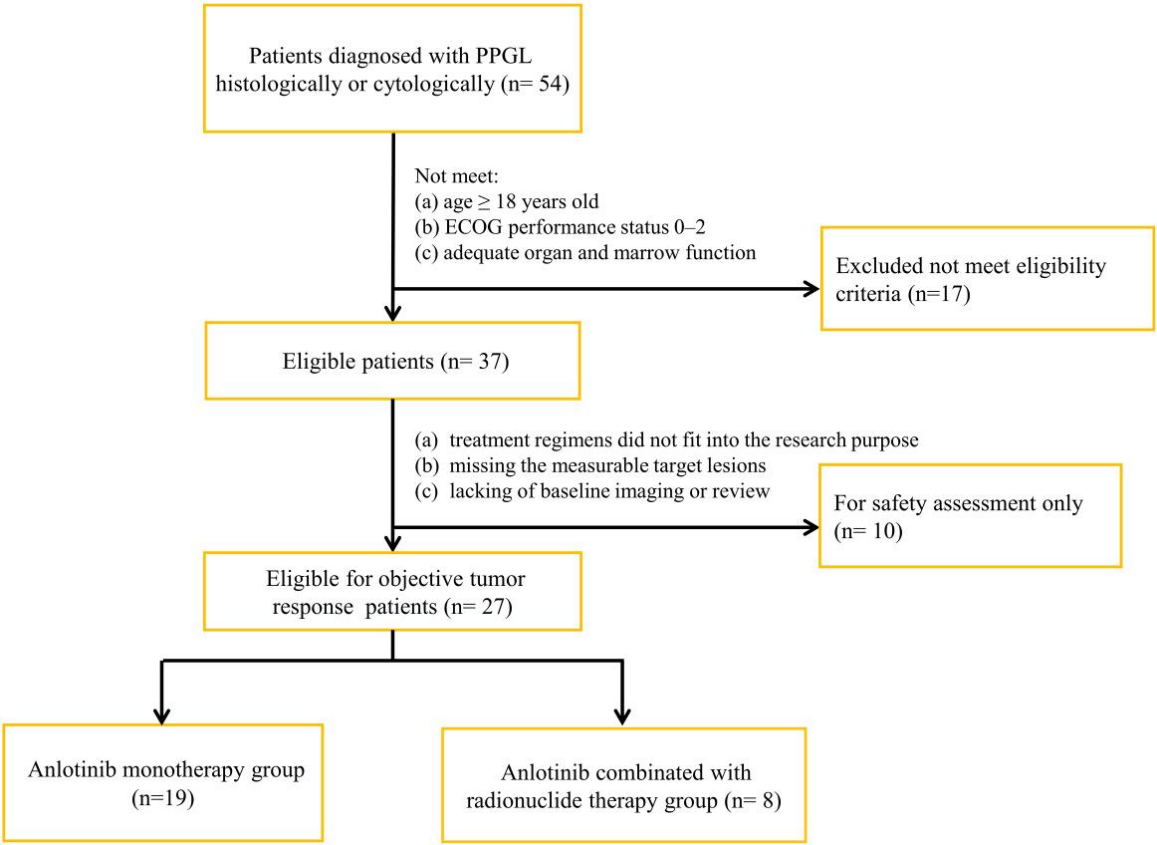
Study flow chart. Abbreviations: ECOG, Eastern Cooperative Oncology Group; PPGL, pheochromocytomas and paragangliomas. The data cutoff date was August 2, 2022.

Patients included in the tumor assessment had at least one radiographically measured lesion according to RECIST version 1.1. Other patients were only included in the safety assessment. All patients were able to complete the questionnaire independently or with assistance. A study flow chart is shown in Fig. 1.

All enrolled patients with catecholamine-secreting PPGL had been assessed by a multidisciplinary team with experience in managing catecholamine-secreting tumors. Patients were receiving a stable antihypertensive medication regimen, and their hypertension was well-controlled before enrollment (systolic ≤ 150 mmHg and diastolic ≤ 90 mmHg, at least 7-14 days of α or β blockers if necessary).

### Positron Emission Tomography–Computed Tomography Imaging and Interpretation

All 37 patients had been previously evaluated by ^131^I-MIBG scintigraphy; 16 showed strong positivity by ^131^I-MIBG imaging, with significantly higher uptake in the tumor than in the liver. Evaluation by ^68^Ga-DOTA-NOC positron emission tomography–computed tomography (PET/CT) had been performed on 28 patients, 24 of whom showed strong positivity in ^68^Ga-DOTA-NOC PET with a Krenning score of more than 3 [28].

LA/M PPGL patients with positive ^68^Ga-DOTA-TATE imaging could also receive ^177^Lu-DOTA-TATE treatment [8, 9], and ^177^Lu-DOTA-TATE and PRRT have been approved for the treatment of somatostatin receptor-positive gastrointestinal and pancreatic neuroendocrine tumors [29]. A previous retrospective study showed that patients treated with ^177^Lu-DOTA-TATE receptor radionuclide therapy had better OS and progression-free survival (PFS) than those treated with conventional ^131^I-MIBG, suggesting that PRRT has good potential for the treatment of metastatic PGL [30]. In our center, PET/CT-based molecular imaging alone or in combination with ^68^Ga-DOTA-TATE/DOTA-NOC and PRRT was performed with ^177^Lu-DOTA-TATE/DOTA-TOC. Despite differences in receptor affinity, there is no convincing evidence supporting that one of these imaging agents is superior to another [31, 32]. Patients who progress rapidly after multi-line therapy and with a large tumor load may be expected to show poor tumor control with a TKI drug alone, according to the clinical experience of the investigators. Combination therapy was therefore considered in this subset of patients.

### Treatment Strategy

In this single-center retrospective study, eligible patients with LA/M PPGL were treated with oral anlotinib alone for a 21-day cycle, starting with 10 to 12 mg once daily on days 1 to 14, followed by a 7-day ceasing period (at least one course of treatment) until disease progression (PD) or intolerability. Eligible patients received anlotinib monotherapy or in combination with PRRT or ^131^I-MIBG radionuclide therapies, as determined by the investigators based on each patient's condition.

Systemic radiotherapy was administered 1 week after the first cycle using anlotinib. PRRT involved the use of ^177^Lu-DOTA-TATE/DOTA-TOC (precursors obtained from ABX, Radeberg, Germany; labeled at the Institute and Department of Nuclear Medicine, Nanjing First Hospital). Eligible patients were treated with 3.77 to 11.1 GBq ^177^Lu-DOTA-TATE/DOTA-TOC via intravenous injection once every 8 to 12 weeks for a maximum of 4 cycles. The injection dosage depended on the location of the primary tumor, metastasis, tumor size, weight, liver and kidney function, and bone marrow function. ^131^I-MIBG therapy was administered with an intravenous treatment-planning (infusion over 30 minutes in an inpatient setting) dose of ^131^I-MIBG (7.4 GBq [200 mCi]) about every 12 weeks. Frequencies of 2 or 3 therapeutic doses a year were considered acceptable and the cumulative therapeutic dose was 29.6 to 37 GBq (800-1000 mCi).

### Efficacy Assessment

Data on hematologic parameters, blood pressure, and catecholamine levels were collected at baseline, 3 months, 6 months, and so on. Computed tomography (CT), magnetic resonance imaging, or ^68^Ga-DOTA-1-Nal3-octreotide (^68^Ga-DOTA-NOC) PET/CT scans were performed at baseline and within 6 months, and tumor response was assessed according to RECIST 1.1 criteria. Attention was paid to the consistency of the imaging methods for the comparisons.

The primary endpoint was the objective response rate (ORR), defined as partial response (PR) or complete response (CR), and the disease-control rate (DCR), defined as PR, CR, or stable disease (SD). Secondary endpoints included progression-free survival (PFS), duration of response (DOR), and drug safety.

We collected relevant biochemical tumor marker results in the medical records system, including concentrations of catecholamines and their metabolites (epinephrine, norepinephrine, normetanephrine, metanephrine, vanillylmandelic acid, homovanillic acid, and dopamine) in plasma and 24-hour urine, as well as serum chromogranin A. The data were collected at baseline and every 3 months throughout the study period by the same laboratory staff. The baseline data were collected 2 weeks before and after the first medication. Because plasma and urinary catecholamines and their metabolites might vary [33, 34], we only evaluated the overall biomarker response, in which any biomarker level was at least two-fold the upper limit of normal. We defined biomarker CR as normalization of the levels of any of the above biomarkers, and PR as a 50% decrease in marker levels at any time during follow-up.

### Safety Assessment

Any adverse events (AEs) that occurred during treatment, including abnormal clinical symptoms, vital signs, and abnormal laboratory tests, were observed. The clinical features, severity, occurrence time, duration, treatment methods, and follow-up were recorded. The correlations between any AEs and anlotinib treatment were determined by the investigators. The safety of the drug was graded by the National Cancer Institute Common Terminology Criteria for Adverse Events (CTCAE v5.0). The investigators adjusted the dosage according to the grade of AEs with reference to the Anlotinib Adverse Reaction Management Manual. According to the protocol, severe AEs (SAEs) were defined as life-threatening, resulting in death, requiring hospitalization or prolongation of hospitalization, or resulting in significant grade of disability, such as hypertensive crisis.

### Statistical Analysis

Normally distributed variables, such as age, were presented as mean ± standard deviation. Other baseline data and AEs were recorded as absolute numbers or percentages. Survival rates, such as PFS and DOR, were estimated by Kaplan–Meier survival curves. A two-tailed *P* value < .05 was considered statistically significant. Statistical analysis was performed using SPSS version 24.0 software (IBM Corp., Armonk, NY, USA).

## Results

### Patient Characteristics

#### Basic characteristics

A total of 37 patients were finally enrolled in this study. The demographic data and clinical characteristics of patient are summarized in Table 1, with 15 cases being diagnosed as PCC and 22 as PGL, for a median follow-up time of 13.58 months (range, 1.13-25.63 months). The median age of the PCC group was older than that of the PGL group. One patient had locally advanced disease and 36 had metastatic status in the liver (n = 12 cases), lung (16), lymph node (29), bone(28), and other metastatic lesions(5), including subcutaneous and breast metastases. In this study, the patients had previously undergone various treatment methods, including chemotherapy, ^131^I-MIBG, PRRT, surgical operation, somatostatin receptor agonist (SSA), everolimus, other TKIs, tumor lesion interventional surgery, and radiotherapy. The number of treatment lines ranged from the first to the third and above, indicating that the patients had exhausted most standard treatment options. However, researchers still found clear evidence of disease progression despite the previous treatment attempts. Twenty-five patients received anlotinib monotherapy and the remaining 12 cases received combination regimens, including PRRT (150-281 mCi, for 1 to 4 cycles) in 7 patients, ^131^I-MIBG (200 mCi, 1-4 cycles) in 2 patients, chemotherapy (eg, platinum and temozolomide) in 2, and immunotherapy (pembrolizumab) in 1 patient.

**Table 1.** Patient characteristics (n = 37)

Characteristics	Total (%)
N	37
Age (mean)	47.05 ± 13.41
Gender, M/F	21/16 (56.76/43.24)
Diagnosis (n)	
PCC	15 (40.54)
PGL	22 (59.46)
Metastatic sites	
Lymph node	29 (78.38)
Liver	12 (32.43)
Lung	16 (43.24)
Bone	27 (72.97)
Others	5 (13.51)
Stage	
I	2 (5.41)
II	14 (37.84)
III	3 (8.11)
IV	10 (27.03)
NE	7 (18.92)
SDH mutation	7 (18.92)
RET	1 (2.70)
VHL	1 (2.70)
BRAF	1 (2.70)
No meaning	3 (8.11)
MIBG status	
Positive	9 (24.32)
Weakly positive	7 (18.92)
Negative	11 (29.73)
Unknown	9 (24.32)
Previous treatment lines (n)	
1	15 (40.54)
2	11 (29.73)
3 or further	10 (27.03)
Secretory status	
Nonsecretory	4 (10.81)
Secretory	31 (83.78)
Unclear	2 (5.41)

Qualitative data are expressed as numbers followed by percentages in parentheses; continuous data are expressed as mean ± standard deviation. Abbreviations: MIBG, meta-iodobenzylguanidine; PCC, pheochromocytoma; PGL, paraganglioma; SDH, succinate dehydrogenase.

The median duration of treatment was 13.05 months (range, 1.07-25.33 months). The starting dosage for 34 patients was 12 mg once daily, and 3 patients started at 10 mg once daily (the investigators determined that these 3 patients had relatively large tumor burdens, significant symptoms, and hypertensive crisis due to hormone release).

#### Genetic mutations

Genetic testing results were available for 13 patients and showed *SDHB* mutations in 5 patients, *SDHD*, *SDHA*, *RET*, *VHL*, and *BRAF* mutations in 1 patient each, and no meaningful mutations in the other 3 patients. The time from pathological diagnosis to inclusion was 26.11 ± 33.072 months. Previously, 16, 11, and 10 patients had received first-line, second-line, and third-line and above treatments, respectively. Previous treatments included surgical resection of primary or metastatic sites in 29 patients, PRRT in 7, chemotherapy in 6, ^131^I-MIBG in 6, and other TKI drugs (sunitinib) in 3.

### Treatment Outcomes

#### Objective tumor response

**Figure 2.**
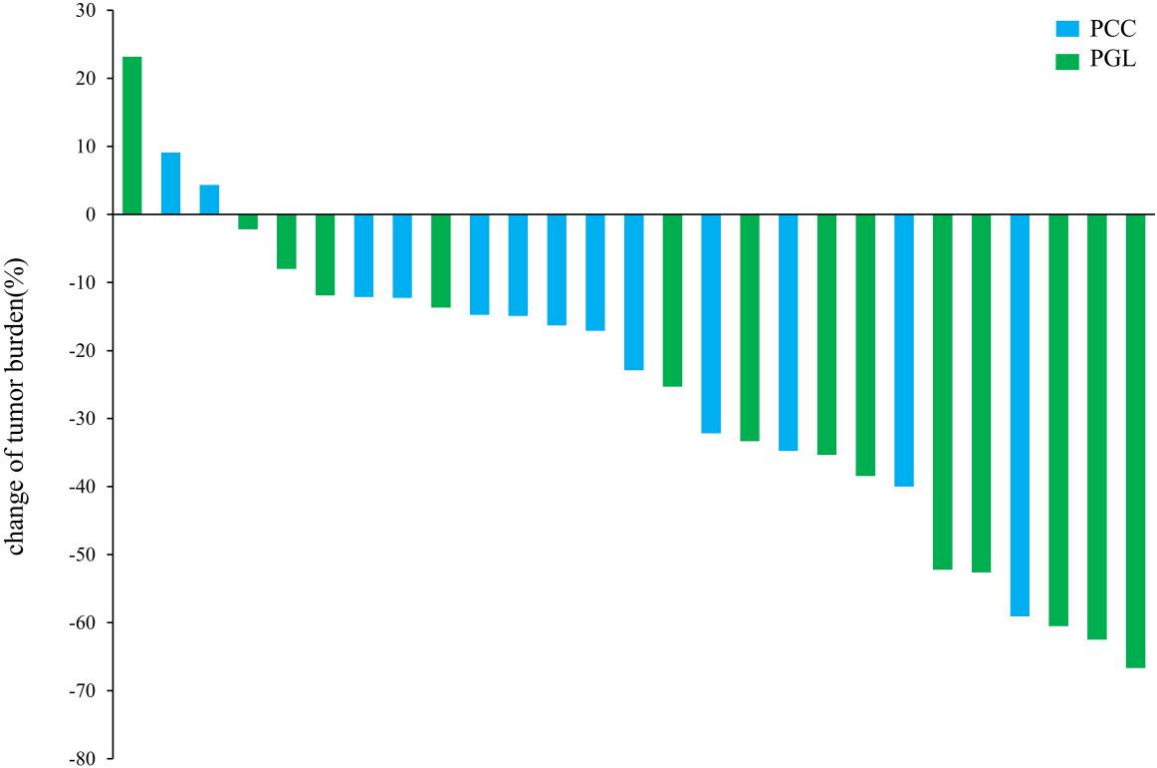
Waterfall plot of best tumor response by RECIST 1.1. Each bar represents the tumor response of each individual patient, there were 12 PR patients, 14 SD patients, and 1 PD patients. Abbreviations: PCC, pheochromocytomas; PGL, paragangliomas.

**Figure 3.**
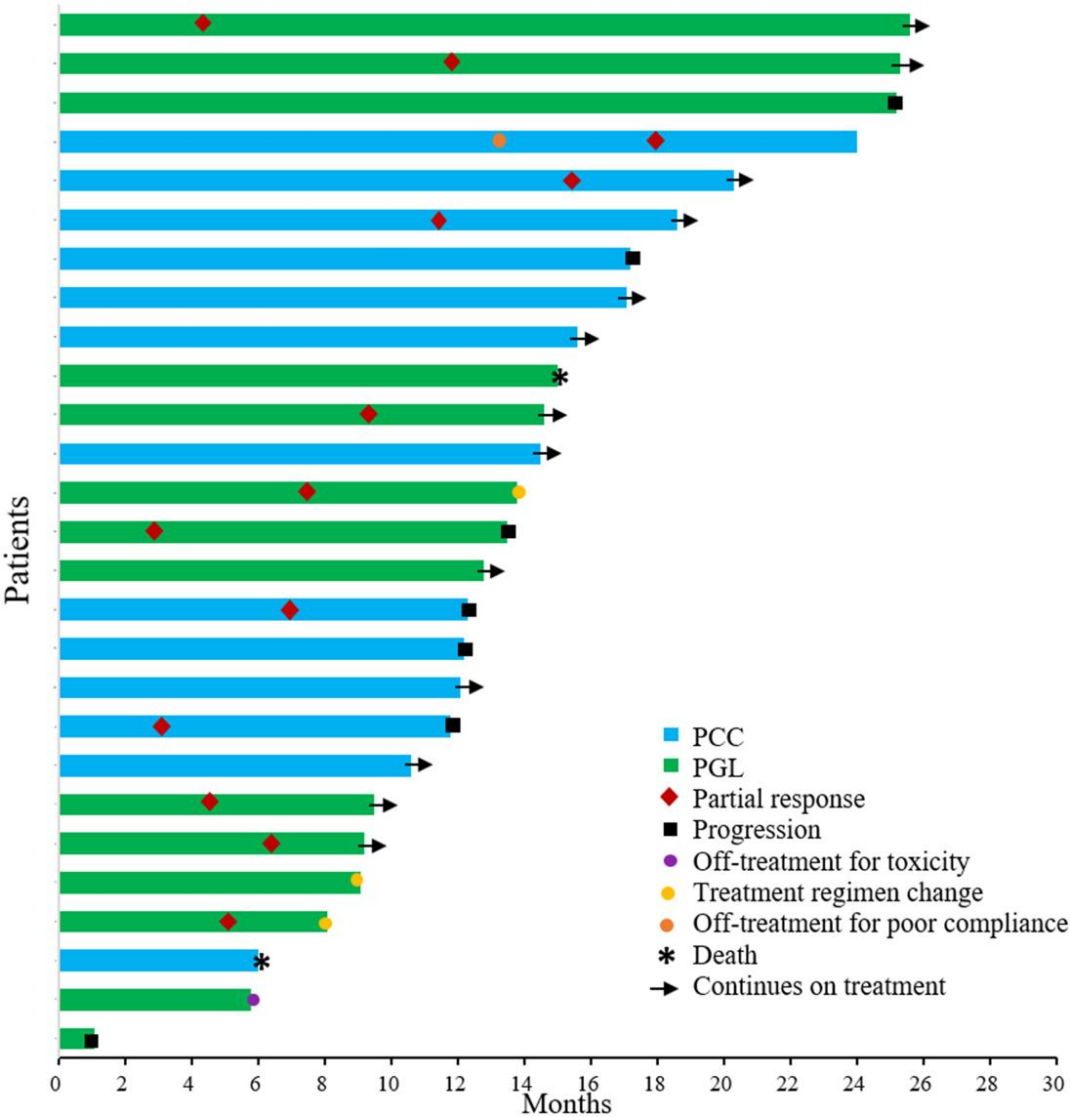
Swimmer plot of duration of tumor response (DOR) in evaluable patients. By the time of data cutoff, 14 patients had a PFS event: 2 dead, 7 had PD, 4 requested a treatment change, and 1 discontinued because of adverse events. 13 patients had SD for up to 9.2 months. The median PFS time was 12.3 months. the longest periods of tumor control were noted in 2 patients with PGL, 1 had a retroperitoneal PGL accompanied by multiple lymph nodes and right mandible metastases, who had PR at the fourth month and the tumor continued to retreat subsequently for up to 25.6 months through treatment with anlotinib combined with ^131^I-MIBG. The other patient had a head and neck PGL (lateral to the common carotid artery in the supraclavicular region) that was metastatic to bone, multiple lymph nodes, she achieved a PR and continued tumor regression after 25.3 months of anlotinib combined with PRRT therapy.

**Figure 4.**
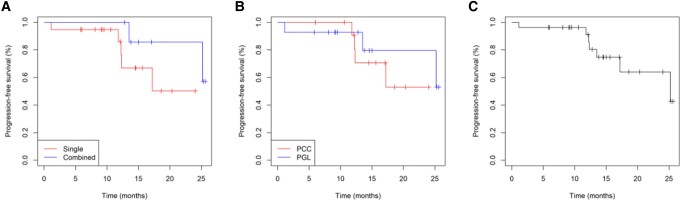
Kaplan-Meier plot of progression-free survival through to August 2, 2022. A, PFS comparisons in anlotinib monotherapy group and anlotinib combination therapy group. B, PFS comparisons in PCC patients and PGL patients. C, PFS in all patients.

**Figure 5.**
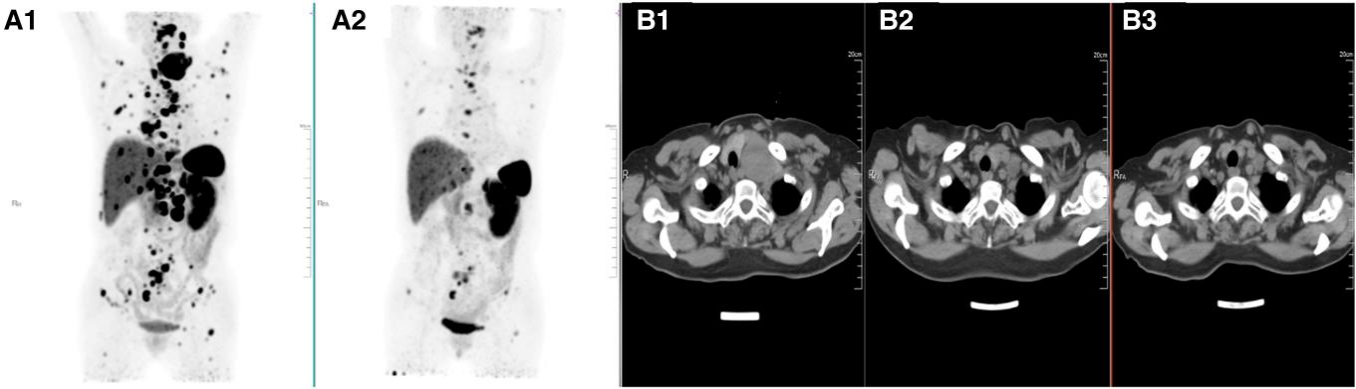
Baseline and review imaging in an anlotinib-treated female patient (aged 57 years old) with retroperitoneal paraganglioma accompanied by metastases in liver, bone, breast, and multiple lymph node. (A1, A2) Maximum intensity projection of 68Ga-DOTA-NOC PET/CT of baseline images and images after 8 months treatment; (B1-B3) CT images of metastases of left cervical lymph node of images on baseline, after 4 months treatment, after 8 months treatment.

Twenty-seven patients were included in the tumor response evaluation, comprising 19 patients in the anlotinib monotherapy group and 8 in the combination radionuclide regimen group (anlotinib combined with PRRT/MIBG). The other 10 patients were only considered for the safety assessment because their treatment regimens did not meet the requirements of the investigators, they did not have measurable target lesions, or they lacked baseline imaging or review data. Figure 2 illustrates the best tumor response by RECIST 1.1. Each bar in the waterfall plot represents the tumor response for an individual patient. Of the 27 patients with evaluable disease, 12 (44.44%) achieved PR, 14 (51.85%) achieved SD lasting > 12 weeks, and no patients had CR. One patient had rapid PD 1 month after enrollment. In the anlotinib monotherapy group, 8 patients (42.1%) achieved PR and 10 patients (52.6%) achieved SD, while in the combination anlotinib/radionuclide regimen group, 4 patients (50%) achieved PR and 4 patients (50%) achieved SD. The ORR was 44.44% (95% CI, 24.4%-64.5%) and the DCR was 96.29% (95% CI, 88.7%-100%). The ORRs for patients with PCC and PGL were 30.77% (95% CI, 17%-59.8%) and 57.14% (95% CI, 27.5%-86.8%), respectively. The time to response and DOR for each evaluable patient are summarized in Fig. 3. At the time of data cutoff (August 2, 2022), the median PFS was 25.2 months (95% CI, 17.2 months to not reached). Nine patients had a PFS event, including 2 deaths and 7 PD, and 5 patients discontinued treatment, of whom 3 requested a treatment change, 1 discontinued the treatment of his own accord, and 1 discontinued because of CTCAE grade 3 bleeding. Thirteen patients had SD for > 9.2 months, and 3 patients (11.11%) died. PFS was lower in the anlotinib monotherapy group than in the anlotinib combination therapy group, but the difference was not significant (*P* = .2) (Fig. 4A). PFS was lower in the PGL group than in the PCC group, but the difference was not significant (*P* = .5) (Fig. 4B). Baseline and review imaging results for a patient with retroperitoneal paraganglioma accompanied by liver, bone, breast, and multiple lymph node metastases are shown in Fig. 5. The patient received first-line surgical treatment for the primary lesion and PRRT therapy, but she still had PD. This patient then received anlotinib monotherapy, with PR.

A patient with PCC accompanied by lung and lymph node metastases had PD after successive resection of the primary and pulmonary lesions, but both lesions regressed to SD after subsequent anlotinib monotherapy for 13.3 months (Fig. 3, fourth bar from the top). The patient decided to stop all antitumor treatment, but a review of his lesion 5 months later (18.8 months) showed a further reduction to about 60% of the original size, indicating that anlotinib had an antitumor effect for PCC even after stopping the medication.

#### Biochemical tumor marker response

Overall, in 29 patients who had evaluable statistics for biochemical tumor marker response, 24 patients (82.5%) had at least one biomarker of diagnostic significance at baseline, with each patient having a different biochemical marker. Specifically, the biochemical CR and PR rates were 20.83% (5/24) and 37.5% (9/24), respectively, the overall response rate was 58.33% (14/24), and the response duration was 30.89 ± 15.85 months. The overall response rate for urinary norepinephrine was 33.33% (3/9) and the response duration was 26.15 ± 20.94 months; the equivalent results for plasma normetanephrine were an overall response rate of 37.5% (3/8) and response duration of 35.81 ± 18.83 months, and for chromogranin A, overall response rate of 47.06% (8/17) and 28.52 ± 19.61 months. The specific hormone profile of each patient is listed in Supplementary Material 2 [35].

### Adverse Events

All patients were enrolled in the safety assessment. All grade 3 to 4 and grade 1 to 2 AEs that occurred in more than 2 patients were suspected to be related to anlotinib (Table 2). Few severe toxicities were observed, and the most common AEs included malaise (25 patients, 67.57%), hypertension (25 patients, 67.57%), palmar–plantar erythrodysesthesia syndrome (PPES) (24 patients, 64.86%), and oral mucositis (20 patients, 54.05%). The most common grade 3 to 4 AEs were hypertension (23 patients, 62.16%) and PPES (3 patients, 8.11%). During the treatment, 5 patients (16.22%) had their dosage decreased to 10 mg, including 2 (5.41%) who changed to 8 mg due to intolerability. The main reasons for the dosage reduction were diarrhea in 2 patients and hypertension, cough, and bleeding in 1 patient. One patient (2.70%) eventually discontinued treatment because of CTCAE grade 3 bleeding, despite dosage reduction to 8 mg. There were no treatment-related SAEs.

**Table 2.** Analysis of adverse events suspected to be related to anlotinib (n = 37)

Adverse events	Grade 1-2 (n, %)	Grade 3-4 (n, %)	Total (n, %)
Hypertension	2 (5.41%)	23 (62.16)	25 (67.57)
Malaise	24 (64.86)	1 (2.70)	25 (67.57)
PPES	21 (56.76)	3 (8.11)	24 (64.86)
Oropharyngeal pain	19 (51.35)	1 (2.70)	20 (54.05)
Hoarseness	17 (45.95)		17 (45.95)
Diarrhea	12 (32.43)	2 (5.41)	14 (37.84)
Bleeding	7 (18.92)	1 (2.70)	8 (21.62)
Hypothyroidism	7 (18.92)		7 (18.92)
Abnormal blood lipids	6 (16.22)		6 (16.22)
Irregular menstruation	5 (31.25)		5 (31.25)*^a^*
Pain	4 (10.81)	1 (2.70)	5 (13.51)
AST/ALT increased	3 (8.11)		3 (8.11)
Proteinuria	3 (8.11)		3 (8.11)
Cough	3 (8.11)		3 (8.11)
Neutropenia	2 (5.41%)		2 (5.41%)
Thrombocytopenia	2 (5.41%)		2 (5.41%)

All grade 3/4 events are reported, only grade 1/2 occurring in more than 2 patients are documented.

Abbreviation: PPES, palmar–plantar erythrodysesthesia syndrome.

^*a*^Indicates that the sample size for female patients for this line of data is n = 16.

## Discussion

To the best of our knowledge, this retrospective study represents the first evaluation of the efficacy and safety of the new multi-TKI anlotinib, both as monotherapy and in combination with radionuclide therapies, in patients with LA/M PPGL. The study achieved its primary endpoint with high ORR (44.44%) and DOR (96.29%) rates and median PFS (25.2 months) by the data cutoff date, with controllable and tolerable AEs. PFS was lower in anlotinib monotherapy group compared with anlotinib in combination with radionuclide regimen group, but the difference was not significant due to the small sample size (*P* = .2). In addition, biochemical tumor marker responses were evident (14/24, 58.33%) in the evaluable patients. These real-world data showed that the antiangiogenic-targeted drug anlotinib had a promising efficacy and favorable safety profile for PPGL treatment, either as monotherapy or in combination with radionuclide therapies. These results warrant further studies of anlotinib.

Regarding the tolerability and compliance, the most common anlotinib-related AE in the current study was hypertension, which was consistent with the use of anlotinib for the treatment of other malignant tumors [24, 36]. However, the incidence (67.57%) in this study was relatively high, possibly due to the characteristics of PPGL itself. Most patients (31/37, 86.49%) in this study had catecholamine-secreting tumors and the baseline blood pressure in most patients was in the high normal range after the use of antihypertensive drugs. Patients were on long-term antihypertensive medication before enrollment and might thus have tolerated the uncomfortable symptoms of hypertension for prolonged periods. Although patients' blood pressure tended to rise significantly after anlotinib therapy, along with hormone release into the bloodstream, only 2 patients required dose reduction due to hypertensive AEs, suggesting that it was relatively safe and controllable after reasonable adjustment of antihypertensive drugs, including dual or triple antihypertensive regimens. Malaise and PPES were also commonly reported AEs (67.57% and 64.86% respectively). Notably, a subgroup analysis showed that the common AEs of hypertension and PPES were correlated with clinical outcomes in patients with non-small cell lung cancer, and patients with these AEs after anlotinib treatment had better median OS [37]. It is possible that the higher incidences of hypertension and PPES in our study may also be related to the higher tumor response rate in the anlotinib treatment group. A major concern with any antiangiogenic treatment is bleeding, such as with bevacizumab [38]. In addition, Jasim et al conducted a phase 2 trial of pazopanib in patients with PPGL and reported that hypertensive crises with associated cardiomyopathy developed in 2 patients with secretory tumors [21]. In our study, there were no CTCAE grade 4 treatment-related SAEs, and only 1 patient discontinued treatment because of bleeding. We attributed this lack of SAEs to the generally favorable physical condition of the subjects, the starting dose selected by the investigators, the timely management of complications, and the intermission period of anlotinib administration (2 weeks on-treatment, followed by ceasing of 1 week).

The treatment of LA/M PPGL remains difficult. Although TKI drugs provide a potentially novel treatment approach, few clinical trials have been completed in patients with PPGL. Sunitinib has been the most well-characterized TKI drug to date. Seventeen patients with metastatic PPGL treated with sunitinib were enrolled in a retrospective study [39], of whom 21.4% attained PR and 35.7% had SD, while 3 patients had to stop treatment because of severe early side-effects. O’Kane et al published the first prospective, randomized, placebo-controlled phase II trial of sunitinib in PPGL, and showed a DCR of 83%, which was lower than our findings [20]. Regarding animal experiments, Moog et al compared the efficacy of several new targeted therapies in SDHB-dependent PPGL mice, and showed that sunitinib was superior to other treatments, including IACS-010759 and talazoparib, with or without temozolomide and belzutifan [40]. We found that anlotinib would be preferable to sunitinib, the most studied TKI drug in LA/M PPGL patients. Specifically, tumor response PR was 21.4% with sunitinib and 44.44% with anlotinib, while SD was 35.7% with sunitinib and 51.85% with anlotinib; In terms of AEs, 3 patients (17.5%) discontinued due to severe AEs and 1 (2.70%) discontinued because of CTCAE grade 3 bleeding in anlotinib. This advantage may be attributed to 2 reasons. First, in our retrospective study, anlotinib combined with radionuclide may have contributed to better tumor regression data, although the difference was not statistically significant. Second, patients received anlotinib on days 1 to 14, followed by cessation for 7 days, while sunitinib was given daily for 4 weeks, followed by 2 weeks observation, for a cycle of 6 weeks, which means that a 7-day cessation period, followed by a shorter treatment cycle may be more tolerable to patients. Although our initial retrospective data suggested that anlotinib was better than sunitinib in terms of efficacy and safety, its use in patients with PPGL needs further validation. Our center has conducted a phase 2 prospective clinical trial of anlotinib in LA/M PPGL and is currently recruiting [41]; this clinical trial could provide more clinical evidence for the effectiveness and safety of anlotinib for the treatment of LA/M PPGL.

Regarding the primary outcome, the longest period of tumor suppression was observed in 3 patients with PGL who received anlotinib in combination with radionuclide therapies (1 patient with ^131^I and 2 with PRRT). In the Kaplan–Meier PFS curve analysis, the PFS curve for the anlotinib combination therapy group gradually pulled away from the curve for the anlotinib monotherapy group; however, the difference was not significant due to the small sample size. There are currently no standardized treatments for patients with LA/M PPGL, but personalized medicine has been considered to have great potential for the treatment and management of PPGL. TKI drugs combined with radionuclide therapies offer a promising research direction for the treatment of PPGL.

This study also had some limitations. A large proportion of patients in this study did not undergo genetic testing for various reasons, and we were therefore unable to analyze the correlation between the genetic results and tumor treatment response, due to insufficient data. In addition, PPGL is a rare tumor and the sample size was therefore relatively small, which also impeded the statistical analysis. This was also a retrospective analysis, with inherent limitations such as selection bias, and further randomized controlled studies are planned to verify the results.

## Conclusion

In this study, we retrospectively analyzed the efficacy and safety of anlotinib in patients with LA/M PPGL. Anlotinib monotherapy or in combination with radionuclide therapies was well-tolerated and produced promising DOR, ORR, and median PFS results. This study thus demonstrates that anlotinib is a potential treatment option for the management of patients with LA/M PPGL.

## Data Availability

Some or all datasets generated during and/or analyzed during the current study are not publicly available but are available from the corresponding author on reasonable request.

## Abbreviations

AE: adverse event
CR: complete response
CTCAE: Common Terminology Criteria for Adverse Events
DCR: disease-control rate
DOR: duration of response
LA/M: locally advanced or metastatic
MIBG: meta-iodobenzylguanidine
ORR: objective response rate
OS: overall survival
PCC: pheochromocytoma
PD: progressive disease
PET/CT: positron emission tomography–computed tomography
PFS: progression-free survival
PGL: paraganglioma
PPES: palmar–plantar erythrodysesthesia syndrome
PPGL: pheochromocytoma/paraganglioma
PRRT: peptide receptor radionuclide therapy
PR: partial response
RECIST: response evaluation criteria in solid tumors
SAE: severe adverse event
SD: stable disease
SDH: succinate dehydrogenase
TKI: tyrosine kinase inhibitor
VEGF: vascular endothelial growth factor
